# Generalizability of toxicity data from oncology clinical trials to clinical practice: toxicity of irinotecan-based regimens in patients with metastatic colorectal cancer

**DOI:** 10.3747/co.v16i6.426

**Published:** 2009-12

**Authors:** V.C. Tam, S. Rask, T. Koru–Sengul, S. Dhesy–Thind

**Affiliations:** * Department of Medicine, McMaster University, Hamilton, ON; † Department of Clinical Epidemiology and Biostatistics, McMaster University, Hamilton, ON; ‡ Department of Oncology, McMaster University, Juravinski Cancer Centre, Hamilton, ON

**Keywords:** Colorectal neoplasms, drug therapy, drug toxicity, clinical trials

## Abstract

**Background:**

The relevance of oncology trial results to clinical practice depends on whether the trial participants are similar to the actual population of patients receiving treatment for the malignancy and whether the patients are treated similarly in both circumstances. Chemotherapy treatments may be more toxic in patients of advanced age and poor performance status—patients typically excluded from clinical trials.

**Methods:**

In a retrospective chart review that included all non-trial patients with metastatic colorectal cancer treated with irinotecan-based chemotherapy from January 2004 to September 2006 at our institution, we quantified and subsequently compared the toxicity rates of the irinotecan regimens in clinical practice with published toxicity rates from corresponding phase iii clinical trials. The primary endpoint was the incidence of grades 3 and 4 diarrhea.

**Results:**

The study included 203 patients, and the irinotecan regimens considered included

- folfiri [irinotecan, leucovorin, 5-fluorouracil (5fu)],
- ifl (bevacizumab, irinotecan, 5fu, leucovorin),
- xeliri (capecitabine, 3-weekly irinotecan), and
- irinotecan monotherapy.

The rates of grades 3 and 4 diarrhea for folfiri, ifl, xeliri, and irinotecan monotherapy in clinical practice were 10%, 15%, 17%, and 21% as compared with 10%, 23%, 20%, and 31% respectively in clinical trials. When only patients meeting trial performance status and age criteria were analyzed, the rates of grades 3 and 4 diarrhea by regimen were 11%, 20%, 19%, and 26% respectively.

**Conclusions:**

Overall, the toxicity rates for folfiri and irinotecan monotherapy in non-trial patients were not statistically different from the rates quoted in published clinical trials.

## 1. INTRODUCTION

Despite that fact that colorectal cancers tend to occur in elderly people, oncology clinical trials have tended to exclude elderly patients in favour of patients who are younger and who have fewer comorbidities and a better performance status 1,2. These population effects—that is, the differences between clinical trial and non-trial patient populations—have led to a general belief that patients enrolled in clinical trials have outcomes that are superior compared with those in patients who are not included 3–5. Trial effects—such as increased frequency of medical imaging, more frequent follow-up visits, and additional attention by clinical trials staff during treatment—may lead to additional benefit for cancer patients participating in clinical trials.

Despite these assumptions, a systematic review has shown that the evidence is insufficient to support a claim of improved outcomes for cancer patients who participate in clinical trials as compared with patients who are not trial participants 6. However, all of the studies included in that review focused on efficacy endpoints such as overall survival and response to treatment; few reported on toxicity.

Irinotecan-based chemotherapy regimens are frequently used in the treatment of metastatic colon cancer, and they have extended the median survival of patients with this disease to 20 months 7. However, this survival benefit is not without risk, because irinotecan regimens are associated with high rates of severe diarrhea 8–10. Although the toxicity rates for folfiri [irinotecan, leucovorin, 5-fluorouracil (5fu)], ifl (bevacizumab, irinotecan, 5fu, leucovorin), xeliri (capecitabine, 3-weekly irinotecan), and irinotecan monotherapy are well documented in publications of phase ii and iii clinical trials, we are unaware of any studies that document the toxicity of these regimens in clinical practice. The objective of the present study was to determine whether chemotherapy toxicity rates in non-trial patients are similar to those quoted in published clinical trials.

## 2. PATIENTS AND METHODS

### 2.1 Study Design

In a retrospective single-institution chart review, the incidence of irinotecan-based chemotherapy toxicity in the palliative treatment of patients with metastatic colorectal cancer was characterized. Toxicity rates in clinical practice were compared with those described in the largest phase iii clinical trial published to date for each regimen. The primary endpoint was the incidence of grades 3 and 4 diarrhea. Secondary endpoints included the incidence of other grade 3 or 4 toxicities, hospital admissions for toxicity related to chemotherapy, dose reductions or delays because of toxicity, discontinuation of chemotherapy secondary to toxicity, and chemotherapy-related mortality.

Patients with metastatic colorectal adenocarcinoma treated with folfiri, ifl, xeliri, or irinotecan monotherapy (Table I) between January 1, 2004, and September 30, 2006, at the Juravinski Cancer Centre (jcc) in Hamilton, Ontario, Canada, were included in the study. Patients were excluded if they received the chemotherapy of interest in a clinical trial, if their pathology was not adenocarcinoma, if they lacked documented metastatic sites, and if they received the chemotherapy of interest at an institution other than the jcc. Charts of the included patients were reviewed for demographic, pathology, treatment, and toxicity data. Two investigators (VCT, SR) completed the data abstraction. Ten percent of the charts were evaluated by both investigators to determine inter-observer variability. Differences in data abstraction were resolved by consensus. The remaining 90% of the charts were then randomly divided between the two investigators for data abstraction.

Toxicities were graded according to the National Cancer Institute Common Terminology Criteria for Adverse Events, version 3.0. If a toxicity grade was not clearly stated in the patient’s chart, the two physician investigators abstracting the data determined a grade based on the information available in each chart according to the previously stated criteria. Toxicity rates observed in patients treated at the jcc were compared with rates from a clinical trial that delivered the identical irinotecan regimen. A separate analysis was conducted to compare younger patients (70 years of age or younger) with elderly patients (older than 70 years of age). A similar comparison was attempted for patients having a good or fair performance status [Eastern Cooperative Oncology Group (ecog) 0–2] with those having a poor performance status (ecog 3–4).

For each regimen included in the study, a representative comparator was chosen from the literature. Where possible, the selected comparator was the largest phase iii trial that used the dose regimen also used at our institution. Our main comparator for folfiri toxicity was the phase iii clinical trial conducted by Colucci *et al.* 11 The toxicity rates for ifl and irinotecan monotherapy were compared with the phase iii trial data published by Saltz *et al.* 9 Unfortunately, phase iii toxicity data were not available for xeliri at the time of our data analysis. The chosen comparator was therefore a phase ii study by Patt *et al.* 12, which was the clinical trial with the largest sample size published up to the time of our data analysis.

### 2.2 Statistical Analysis

Data were recorded and organized using a Microsoft Excel spreadsheet (Redmond, WA, U.S.A.). Statistical analysis was performed using SAS for Windows (version 9.1: SAS Institute, Cary, NC, U.S.A.). Inter-observer variability in data abstraction was assessed by calculating percentage agreement and kappa statistics. Unadjusted and adjusted analyses were both performed. The unadjusted analysis included all non-trial patients; it describes any benefit derived from the combination of population and trial effects. The adjusted analysis excluded all non-trial patients who did not meet the clinical trial age and performance status criteria described in the corresponding comparator trials, effectively eliminating population effects and focusing on trial effects. Tabulations and descriptive statistics were used to analyze the toxicity data. Two-sided hypothesis tests for proportions were performed using the chi-square test or the Fisher exact test when appropriate. A *p* value less than or equal to 0.05 was considered significant.

## 3. RESULTS

### 3.1 Patient Characteristics

**Figure 1:**
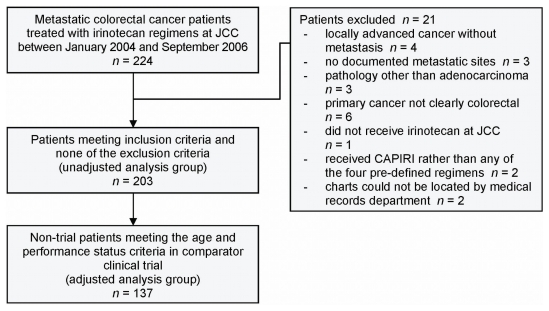
Method of patient selection. jcc = Juravinski Cancer Centre; capiri = capecitabine–irinotecan.

Figure 1 outlines the patient selection process. The study included 203 patients in the unadjusted analysis. Of the 203 patients, 137 (67%) met the age and performance status criteria of their respective comparator clinical trials, and they were used in the adjusted analysis.

Table II outlines the characteristics of the 203 patients. Fifty patients were over the age of 70 years, and 34 of those patients received irinotecan monotherapy. An ecog performance status at the time of therapy was not recorded for 29% of the patients. Of the patients with a clearly described ecog performance status, 95% were classified as better than or equal to 2. At the time of cancer diagnosis, 64% of the patients presented with metastatic disease. Upon initiation of irinotecan treatment, 64% of the patients had two or more metastatic sites. Liver metastases were present in 78% of the patients.

For the charts reviewed in duplicate, agreement was 100% (κ = 1.0) between the two investigators for most of the abstracted data. The exceptions were dose reductions and dose delays. Agreement for the number of dose reductions was 96% (κ = 0.93), and for dose delays, it was 85% (κ = 0.72).

### 3.2 Rates of Toxicity in the Unadjusted Analysis

Table III outlines rates of common toxicities encountered by non-trial patients during treatment with irinotecan regimens. Overall, diarrhea was the most common adverse event, occurring in 61% of all non-trial patients. Grades 3 and 4 diarrhea were reported in 10% of patients receiving folfiri, in 15% receiving ifl, in 17% receiving xeliri, and in 21% receiving irinotecan monotherapy. There was no statistically significant difference in the rates of grades 3 and 4 diarrhea observed in non-trial patients treated with the four irinotecan regimens as compared with the rates observed in patients treated in the comparator clinical trials.

Neutropenia was the second most common toxicity, occurring in 54% of the patients. Grades 3 and 4 neutropenia were reported in 22% of patients receiving folfiri, in 15% receiving ifl, in 11% receiving xeliri, and in 19% receiving irinotecan monotherapy. The rate of grades 3 and 4 neutropenia was significantly higher in the non-trial patients treated with folfiri than in the patients in the study by Colucci *et al.* (*p* < 0.01). For ifl and irinotecan monotherapy, the rates of grades 3 and 4 neutropenia were significantly lower in the non-trial patients than in the patients in the published comparator studies (*p* = 0.01 and *p* = 0.02 respectively). A significantly lower rate of grades 3 and 4 vomiting was also observed for non-trial patients treated with irinotecan monotherapy as compared with patients in the clinical trial by Saltz *et al.* (*p* = 0.05).

Only 9 patients developed febrile neutropenia during the study period. Grade 3 febrile neutropenia was observed in 5% of patients receiving folfiri, 8% receiving ifl, 6% receiving xeliri, and 3% receiving irinotecan monotherapy. No reports of grade 4 febrile neutropenia were found for the patients included in the present study.

### 3.3 Rates of Toxicity in the Adjusted Analysis

Table III also shows toxicity rates resulting from the adjusted analysis. Grades 3 and 4 diarrhea were reported in 11% of patients receiving folfiri, in 20% receiving ifl, in 19% receiving xeliri, and in 26% receiving irinotecan monotherapy. Again, there was no statistically significant difference between the rates of grades 3 and 4 diarrhea observed with the four irinotecan regimens in the non-trial patients and the rates observed in the relevant published clinical trial data.

With the exclusion of non-trial patients who failed to meet the age and performance criteria in the comparator clinical trials, no significant changes in the toxicity rates occurred. For folfiri in the non-trial population, the rates of mucositis and vomiting of any grade remained significantly lower and the rates of neutropenia of any grade and grades 3 and 4 neutropenia remained significantly higher than the rates reported in the clinical trial results. The rates of grades 3 and 4 neutropenia with ifl, vomiting of any grade with xeliri, and grades 3 and 4 vomiting with irinotecan monotherapy remained significantly lower in non-trial patients. The lower rate of grades 3 and 4 neutropenia for non-trial patients treated with irinotecan monotherapy was no longer statistically significant in the adjusted analysis (*p* = 0.12).

### 3.4 Consequences of Chemotherapy Toxicity

Table IV shows the clinical consequences of toxicities from treatment with the four irinotecan regimens. Only selected consequences of chemotherapy toxicity were reported in the publications of the comparator trials, and therefore a systematic comparison was not possible. With the information provided by the comparator clinical trial publications, we found several statistically significant differences. In the unadjusted analysis, the rate of premature discontinuation of ifl treatment secondary to toxicity was 31% in the non-trial patients as compared with 8% in the trial patients (*p* = 0.02). For non-trial patients treated with irinotecan monotherapy, the rate of premature discontinuation of irinotecan chemotherapy in the non-trial patients secondary to toxicity was in the range 24%–26%, which was significantly higher than the 6% quoted in the comparator trial (*p* < 0.01). A similar trend was observed for deaths related to irinotecan monotherapy toxicity, which showed a death rate in non-trial patients of 4%–7% as compared with 1% in the comparator trial (*p* = 0.03).

Overall, there was no clear trend toward an increase or decrease in the rates of toxicity consequences when comparing the unadjusted and adjusted analyses. The highest rates of hospital admission, dose delays, premature discontinuation of irinotecan chemotherapy, and deaths related to irinotecan chemotherapy were seen with ifl as compared with the other three irinotecan regimens. The rate of overall death while on irinotecan chemotherapy was also highest in patients treated with ifl (15%).

### 3.5 Comparison of Toxicity According to Age

The analysis of irinotecan toxicity in younger patients as compared with elderly patients (older than 70 years) showed no significant differences in the rates of diarrhea, mucositis, vomiting, neutropenia, and febrile neutropenia between the two groups. However, admissions for chemotherapy side effects were significantly more frequent in the elderly patients as compared with the younger patients (26% vs. 10%, *p* = 0.02). The incidence of premature discontinuation of irinotecan chemotherapy was also significantly higher in the elderly patients (30% vs. 14%, *p* = 0.02).

### 3.6 Comparison of Toxicity According to Performance Status

In 145 patients, a performance status was clearly defined; data regarding performance status were unavailable in the remaining patient records. Irinotecan toxicity for patients with a good or fair performance status (ecog 0–2) was compared with that for patients with a poor performance status (ecog 3–4). However, only 7 non-trial patients had a poor performance status, and the sample size was therefore too small for any meaningful comparisons to be made.

## 4. DISCUSSION

As compared with patients in published clinical trials, non-trial patients with metastatic colorectal cancer do not appear to experience increased rates of toxicity when treated with irinotecan chemotherapy. The only exception is a higher rate of grades 3 and 4 neutropenia in non-trial patients treated with folfiri: 22% versus 10% in a comparator study (*p* < 0.01). However, that finding might be explained by the abnormally low rate of grades 3 and 4 neutropenia reported in the study by Colucci *et al.* The combined incidence of grades 3 and 4 neutropenia in phase iii clinical trials of folfiri has been noted to be 24%, which is nearly equivalent to the rate in our study population 7.

The rates of several toxicities were actually significantly lower in our non-trial patients, as in the case of vomiting of any grade in the folfiri group and of grades 3 and 4 neutropenia in the ifl group. One possible explanation for these differences is that the documentation of toxicities is poor in the chart notes of non-trial patients as compared with patients on clinical trials. Some of the significantly lower toxicity rates may also be a result of chance, because multiple comparisons were made and sample sizes were small in some groups—particularly for ifl, which is now seldom used in clinical practice, and for xeliri, which is a relatively new regimen.

Peppercorn *et al.* published a systematic review of studies comparing outcomes between trial and non-trial cancer patients 6. In unadjusted analyses, 13 of 21 studies (62%) showed some evidence of improved outcomes in trial patients. In adjusted analyses, where confounding factors were accounted for, 11 of 17 studies (65%) showed better outcomes in the trial patients. However, the overall conclusion by those authors was that the evidence is insufficient to prove that enrolment in cancer clinical trials leads to improved outcomes. It should be noted that nearly all the outcomes examined by studies in the systematic review were efficacy endpoints such as survival and response rate. Because treatment decisions are based both on efficacy and on toxicity of a chemotherapy regimen, our study provides oncologists with important evidence supporting the generalizability of clinical trial toxicity rates to non-trial patients.

Specific age and performance status criteria are prominent in the inclusion criteria for many clinical trials. These factors are important in the analysis of irinotecan toxicity, because age older than 70 years and ecog performance status less than 2 have generally been regarded as risk factors for developing irinotecan toxicity. As a result, several large clinical trials of irinotecan regimens have used an initial dose reduction for all elderly patients 13–15.

With respect to age, it is well documented that patients enrolled in clinical trials tend to be younger than non-trial patients and also younger than the overall population of patients with the malignancy 1,2,4. Differences in age are theoretically important, because medications have different pharmacokinetics in elderly people, who tend to have decreased renal and liver function, lower total body water, and higher amounts of adipose tissue 16,17. In addition, elderly people are generally perceived as having multiple comorbid conditions, limited socioeconomic support, and reduced cognition, which may limit the potential benefit from systemic cancer therapy 17. Despite these theoretic risks, we did not find any significant increase in grades 3 and 4 toxicity rates in non-trial patients older than 70 years. Our findings are supported by several studies that have found irinotecan regimens to be well tolerated in fit elderly patients 18–21. Thus, the current general consensus is that elderly patients fit enough to enrol in clinical trials can be treated with chemotherapy. Frail elderly patients are more likely to suffer adverse outcomes when faced with stressors, and they should not receive systemic chemotherapy. Unfortunately, because of the underrepresentation of elderly patients in cancer clinical trials, the evidence is insufficient to support or oppose the use of chemotherapy in the preponderance of elderly patients who have an intermediate performance status 22.

It has been well established that patients with a poor performance status have a worse prognosis and that they may have reduced response to chemotherapy treatment and increased rates of toxicity 23. As a result, these patients are almost always excluded from clinical trials. Of patients in the present study with a clearly defined performance status, the proportion that were ecog 0 or 1 was 82% among non-trial folfiri patients as compared with 98% among patients in the Colucci *et al.* comparator study. Also, of our non-trial folfiri patients with a clearly determined performance status, 5% had an ecog performance status of 3 and would have been ineligible for the comparator trial. That ineligible proportion may have been even greater, given that performance status was not documented for 26% of the folfiri patients.

The present study has several limitations. Because of the retrospective design, we were unable to mandate rigorous documentation of toxicities for the non-trial patients, possibly leading to underestimation of toxicity rates. Although we attempted to adjust the analysis for the inclusion criteria of the clinical trials, only age and performance status were accounted for. The four comparator trials used numerous exclusion criteria that varied between them, preventing a practical accounting for those criteria in the non-trial population.

## 5. CONCLUSIONS

The folfiri and irinotecan monotherapy regimens do not appear to cause higher rates of toxicity in non-trial patients than in patients treated in clinical trials. However, we advise caution in generalizing clinical trial toxicity data to patients who fail to meet trial inclusion criteria.

## 6. CONFLICT OF INTEREST DISCLOSURE

This study received no funding support.

## Figures and Tables

**TABLE I:** Chemotherapy regimens

Regimen name	Chemotherapy agents	Starting dose	Schedule per cycle
folfiri	Irinotecan	180 mg/m^2^	Once every 2 weeks
	5-Fluorouracil	2400 mg/m^2^ 24-h intravenous infusion	With irinotecan
	Leucovorin	400 mg/m^2^	With irinotecan
ifl	Irinotecan	80–125 mg/m^2^ or 180 mg/m^2^	Once weekly for 4–6 weeks Once every 2 weeks
	5-Fluorouracil	2400 mg/m^2^ intravenous bolus	With irinotecan
	Leucovorin	400 mg/m^2^	With irinotecan
xeliri	Irinotecan	250 mg/m^2^	Once every 3 weeks
	Capecitabine	1000 mg/m^2^ orally	Twice daily for 14 days
Irinotecan monotherapy	Irinotecan	80 mg/m^2^ or 125 mg/m^2^	Once weekly for 4 weeks Once weekly for 4 weeks

**TABLE II:** Patient characteristics

Characteristic	Regimen group			
	folfiri (*n*=102)	ifl (*n*=13)	xeliri (*n*=18)	Irinotecan monotherapy (*n*=70)
Age (years)^a^				
Mean ± standard deviation	59±10	59±11	58±14	68±9
Median	61	54	60	70
Range	29–84	46–83	19–73	44–84
Age > 70 years [*n* (%)]	10 (10)	2 (15)	4 (22)	34 (49)
Sex [*n* (%)]				
Female	46 (45)	6 (46)	8 (44)	25 (36)
Male	56 (55)	7 (54)	10 (56)	45 (64)
ecog performance status [*n* (%)]				
0	35 (34)	3 (23)	9 (50)	21 (30)
1	26 (26)	2 (15)	6 (33)	15 (21)
2	10 (10)	0 (0)	1 (6)	10 (14)
3	4 (4)	0 (0)	0 (0)	3 (4)
Not available	27 (26)	8 (62)	2 (11)	21 (30)
Site of primary tumour [*n* (%)]				
Colon	62 (61)	7 (54)	14 (78)	39 (56)
Rectum	37 (36)	5 (38)	4 (22)	31 (44)
Not available	3 (3)	0 (0)	0 (0)	0 (0)
Metastatic at diagnosis [*n* (%)]				
Yes	60 (59)	9 (69)	13 (72)	47 (67)
No	42 (41)	4 (31)	5 (28)	23 (33)
Pathology [*n* (%)]				
Well differentiated	7 (7)	1 (8)	0 (0)	2 (3)
Moderately differentiated	73 (72)	9 (69)	15 (83)	45 (64)
Poorly differentiated	20 (20)	0 (0)	1 (6)	17 (24)
Undifferentiated	0 (0)	0 (0)	1 (6)	0 (0)
Not available	2 (2)	3 (23)	1 (6)	6 (9)
Metastatic sites [*n* (%)]				
1	31 (30)	6 (46)	6 (33)	27 (39)
2	40 (39)	4 (31)	7 (39)	28 (40)
>2	31 (30)	3 (23)	5 (28)	15 (21)
Liver involvement [*n* (%)]				
Yes	77 (76)	9 (69)	15 (83)	57 (81)
No	25 (24)	4 (31)	3 (17)	13 (19)

a At date of first irinotecan treatment.

folfiri = irinotecan, leucovorin, 5-fluorouracil; ifl = bevacizumab, irinotecan, 5-fluorouracil, leucovorin; xeliri = capecitabine, 3-weekly irinotecan; ecog = Eastern Cooperative Oncology Group.

**TABLE III:** Toxicity comparisons by regimen

Regimen and toxicity	Unadjusted analysis			Adjusted analysis	
	Trial pts [*n* (%)]	Non-trial pts [*n* (%)]	*p* Value	Non-trial pts [*n* (%)]	*p* Value
*folfiri*	(*n=*178)^a^	(*n=*102)		(*n=*70)	
Diarrhea					
Any grade	113 (63)	55 (54)	0.07	39 (56)	0.26
Grades 3 and 4	18 (10)	10 (10)	1.00	8 (11)	0.80
Mucositis					
Any grade	63 (35)	22 (22)	<0.01	16 (23)	0.04
Grades 3 and 4	2 (1)	1 (1)	1.00	1 (1)	1.00
Vomiting					
Any grade	128 (71)	26 (26)	<0.01	19 (27)	<0.01
Grades 3 and 4	8 (4)	3 (3)	1.00	2 (3)	0.93
Neutropenia					
Any grade	80 (45)	57 (56)	<0.01	43 (61)	<0.01
Grades 3 and 4	17 (10)	23 (22)	<0.01	16 (23)	<0.01
Febrile neutropenia					
Any grade	na	5 (5)	—	4 (6)	—
Grades 3 and 4	na	5 (5)	—	4 (6)	—
*ifl*	(*n=*225)^b^	(*n=*13)		(*n=*5)	
Diarrhea					
Any grade	na	8 (62)	—	4 (80)	—
Grades 3 and 4	na (23)	2 (15)	0.81	1 (20)	1.00
Mucositis					
Any grade	na	3 (23)	—	1 (20)	—
Grades 3 and 4	na (2)	0 (0)	1.00	0 (0)	1.00
Vomiting					
Any grade	na	2 (15)	—	1 (20)	—
Grades 3 and 4	na (10)	0 (0)	0.53	0 (0)	1.00
Neutropenia					
Any grade	na	9 (69)	—	4 (80)	—
Grades 3 and 4	na (54)	2 (15)	0.01	0 (0)	0.04
Febrile neutropenia					
Any grade	na (7)	1 (8)	1.00	1 (20)	0.61
Grades 3 and 4	na	1 (8)	—	1 (20)	—
*xeliri*	(*n=*51)^c^	(*n=*18)		(*n=*16)	
Diarrhea					
Any grade	na (78)	14 (78)	1.00	12 (75)	0.96
Grades 3 and 4	na (20)	3 (17)	1.00	3 (19)	1.00
Mucositis					
Any grade	na	1 (6)	—	1 (6)	—
Grades 3 and 4	na	0 (0)	—	0 (0)	—
Vomiting					
Any grade	na (61)	6 (33)	0.03	5 (31)	0.03
Grades 3 and 4	na (17)	0 (0)	0.07	0 (0)	0.10
Neutropenia					
Any grade	na (37)	6 (33)	0.98	5 (31)	0.85
Grades 3 and 4	na (25)	2 (11)	0.27	2 (12)	0.39
Febrile neutropenia					
Any grade	na	1 (6)	—	1 (6)	—
Grades 3 and 4	na	1 (6)	—	1 (6)	—
*Irinotecan monotherapy*	(*n=*223)^b^	(*n=*70)		(*n=*46)	
Diarrhea					
Any grade	na	46 (66)	—	31 (67)	—
Grades 3 and 4	na (31)	15 (21)	0.10	12 (26)	0.58
Mucositis					
Any grade	na	5 (7)	—	3 (6)	—
Grades 3 and 4	na (2)	1 (1)	1.00	1 (2)	1.00
Vomiting					
Any grade	na	19 (27)	—	12 (26)	—
Grades 3 and 4	na (12)	3 (4)	0.05	1 (2)	0.04
Neutropenia					
Any grade	na	38 (54)	—	29 (63)	—
Grades 3 and 4	na (31)	13 (19)	0.02	9 (19)	0.12
Febrile neutropenia					
Any grade	na (6)	2 (3)	0.44	2 (4)	0.94
Grades 3 and 4	na	2 (3)	—	2 (4)	—

a Colucci *et al.* 11

b Saltz *et al.* 9

c Patt *et al.* 13

na = not available from published clinical trial article.

**TABLE IV:** Consequences of chemotherapy toxicity

Consequence of toxicity	Patients [*n* (%)]							
	folfiri		ifl		xeliri		Irinotecan monotherapy	
	Adjusted		Adjusted		Adjusted		Adjusted	
	No (*n*=102)	Yes (*n*=70)	No (*n*=13)	Yes (*n*=5)	No (*n*=18)	Yes (*n*=16)	No (*n*=70)	Yes (*n*=46)
One or more admissions for irinotecan chemo-therapy side effects	11 (11)	8 (11)	3 (23)	1 (20)	1 (6)	1 (6)	13 (19)	10 (22)
Dose delays secondary to irinotecan toxicity	43 (42)	31 (44)	9 (69)	4 (80)	6 (33)	6 (38)	30 (43)	18 (39)
Dose reductions secondary to irinotecan toxicity	40 (39)	29 (41)	3 (23)	1 (20)	8 (44)	7 (44)	35 (50)	23 (50)
Premature discontinuation of irinotecan chemo- therapy secondary to toxicity	12 (12)	9 (13)	4 (31)	2 (40)	3 (17)	2 (12)	17 (24)	12 (26)
Deaths while still on irinotecan chemotherapy	9 (9)	4 (6)	2 (15)	0 (0)	0 (0)	0 (0)	6 (9)	3 (7)
Deaths related to irinotecan chemotherapy toxicity	0 (0)	0 (0)	1 (8)	0 (0)	0 (0)	0 (0)	3 (4)	3 (7)
